# Zolmitriptan: A Novel Portal Hypotensive Agent Which Synergizes with Propranolol in Lowering Portal Pressure

**DOI:** 10.1371/journal.pone.0052683

**Published:** 2013-01-16

**Authors:** Mercedes Reboredo, Haisul C. Y. Chang, Roberto Barbero, Carlos M. Rodríguez-Ortigosa, Francisco Pérez-Vizcaíno, Asunción Morán, Mónica García, Jesús M. Banales, Norberto Carreño, Félix Alegre, Ignacio Herrero, Jorge Quiroga, Jesús Prieto, Bruno Sangro

**Affiliations:** 1 Centro de Investigación Biomédica en Red de Enfermedades Hepáticas y Digestivas (CIBERehd), Pamplona, Spain; 2 Division of Hepatology and Gene Therapy, Center for Applied Medical Research (CIMA), University of Navarra, Pamplona, Spain; 3 Department of Pharmacology, Universidad Complutense, Madrid, Spain; 4 Centro de Investigación Biomédica en Red de Enfermedades Respiratorias (CIBERES), Madrid, Spain; 5 Departamento de Fisiología y Farmacología, Universidad de Salamanca, Salamanca, Spain; 6 IKERBASQUE, Division of Hepatology and Gastroenterology, Biodonostia Research Institute – Donostia Universitary Hospital, University of Basque Country UPV/EHU, San Sebastián, Spain; 7 Liver Unit, Clínica Universitaria de Navarra, Pamplona, Spain; Medical University Graz, Austria

## Abstract

**Objective:**

Only a limited proportion of patients needing pharmacological control of portal hypertension are hemodynamic responders to propranolol. Here we analyzed the effects of zolmitriptan on portal pressure and its potential interaction with propranolol.

**Methods:**

Zolmitriptan, propranolol or both were tested in two rat models of portal hypertension: common bile duct ligation (CBDL) and CCl4-induced cirrhosis. In these animals we measured different hemodynamic parameters including portal venous pressure, arterial renal flow, portal blood flow and cardiac output. We also studied the changes in superior mesenteric artery perfusion pressure and in arterial wall cAMP levels induced by zolmitriptan, propranolol or both. Moreover, we determined the effect of splanchnic sympathectomy on the response of PVP to zolmitriptan.

**Results:**

In both models of portal hypertension zolmitriptan induced a dose-dependent transient descent of portal pressure accompanied by reduction of portal flow with only slight decrease in renal flow. In cirrhotic rats, splanchnic sympathectomy intensified and prolonged zolmitriptan-induced portal pressure descent. Also, propranolol caused more intense and durable portal pressure fall when combined with zolmitriptan. Mesenteric artery perfusion pressure peaked for about 1 min upon zolmitriptan administration but showed no change with propranolol. However propranolol enhanced and prolonged the elevation in mesenteric artery perfusion pressure induced by zolmitriptan. *In vitro* studies showed that propranolol prevented the inhibitory effects of β2-agonists on zolmitriptan-induced vasoconstriction and the combination of propranolol and zolmitriptan significantly reduced the elevation of cAMP caused by β2-agonists.

**Conclusion:**

Zolmitriptan reduces portal hypertension and non-selective beta-blockers can improve this effect. Combination therapy deserves consideration for patients with portal hypertension failing to respond to non-selective beta-blockers.

## Introduction

Portal hypertension is the main complication of liver cirrhosis. It develops in the majority of patients and is responsible for massive gastrointestinal bleeding from ruptured gastro-esophageal varices. Portal hypertension also plays a key role in other complications of cirrhosis, such as ascites, hepatorenal syndrome and hepatic encephalopathy [1]. Portal hypertension is associated with the development of hyperdynamic circulation as result of intense splanchnic vasodilation and increased splanchnic blood flow [2]. Pharmacological therapy of this condition is basically aimed at correcting the increased portal blood inflow using splanchnic vasoconstrictors. Some of these drugs, such as non-selective beta-blockers (NSBB), can be given orally and are therefore appropriate for long-term administration. Propranolol is the drug most commonly used in portal hypertensive patients. Propranolol seems to act by limiting the splanchnic vasodilating effects resulting from the enhanced β2-adrenoceptor drive present in cirrhotic patients owing to increased sympathetic tone [3]. Others vasoconstrictors, such as somatostatin or terlipressin, require parenteral administration and are only used for short-term administration, as in the treatment of acute variceal hemorrhage [1]. Unfortunately, only 37% of patients are hemodynamic responders to propranolol [4] and therefore new treatment regimes are much needed. Triptans are agonists of serotonin 5-HT_1B/1D/1F_ receptors commonly used to treat migraine. Zolmitriptan, a selective 5-HT_1B/1D_ receptor agonist [5] was the first of a second generation of triptans. Importantly, 5-HT_1B/D_ receptors are expressed in human superior mesenteric artery where they mediate a potent vasoconstrictor effect [6]. The aim of our study was to evaluate whether zolmitriptan may reduce experimental portal hypertension, to analyze the pharmacological interaction between zolmitriptan and propranolol and to determine if zolmitriptan can provide better control of PVP in combination with propranolol.

## Materials and Methods

### Animals Care

Male Sprague-Dawley rats were purchased from Harlan Laboratories (Italy). Rats weighing 300–350 g were used. Animals were maintained under standard conditions in environmentally controlled animal facilities. All procedures were approved by the by the University of Navarra Animal Care Committee (approval ID: 013/10 and 096/10 ). All surgery was performed under inhalational anesthesia, and all efforts were made to minimize suffering. Sacrifice was performed during deep anesthesia.

### Induction of Portal Hypertension

#### a) Common bile duct ligation (CBDL)

Under isoflurane anesthesia (Abbott Laboratories, UK) the bile duct was doubly ligated with 3–0 non absorbable silk thread (ETHICON, USA) and then resected between the two ligatures. Ketoprofeno (Merial, France) was administered for analgesic purposes. Vitamin k (Roche, Spain) and enrofloxacin (Laboratorios Ovejero, Spain) were administered after surgery to improve survival. Two or four weeks after surgery rats were used in hemodynamic studies.

#### b) Liver cirrhosis was provoked by repeated oral administration of CCl_4_ [7]

Rats were given phenobarbital (Kern Pharma, Spain) in their drinking water throughout all the period of liver cirrhosis induction. One week after, CCl4 (Sigma-Aldrich, Spain) diluted in water was administered orally by gavage. The initial dose was 20 µl and subsequent administrations, on a weekly basis, were dose-adjusted based on changes in body weight. After 12–20 weeks of CCl4 gavage, administration of CCl4 and phenobarbital was stopped and studies were performed one week later.

### Hemodynamic Determinations

All animals were fasted 12 hours before surgery. Measurements were conducted under general inhalation anesthesia (1.5% isoflurane). Continuous anesthetic administration was used to reduce oscillations in drug concentration. Furthermore, isoflurane has been shown to preserve hemodynamic parameters in cirrhotic rats [8]–[9]. A homeothermic blanket (Panlab, Spain) was used to maintain body temperature at 37°C. Portal vein was cannulated via the ileocolic vein with a catheter (22G BD, Spain) connected to a physiological pressure transducer (AD instruments, Australia) to monitor portal venous pressure (PVP). A perivascular ultrasonic transit-time flowprobe (0.7PSB for arterial renal flow (ARF), and 2PSB for portal blood flow (PBF)) (Transonic Systems, USA) was used to measure flow (ml/min). The flow probe was connected to a small animal flowmeter (Transonic Systems, USA). Systemic arterial pressure (MAP) was measured from a fluid-filled catheter placed in a carotid artery (20G). Cardiac output (CO) was determined using ultrasound velocity dilution method as previously described [10]. Blood pressure and flow were registered on a multichannel computer based recorder (AD instruments, Australia). Heart rate (HR) (beats/min) was obtained from arterial blood flow recordings. Systemic vascular resistance (SVR) was calculated as: MAP/CO. Basal measurements were obtained approximately 20 minutes after probe implantation to allow stabilization. Femoral vein was catheterized (24G) for drug infusion.

### Determination of Mesenteric Artery Perfusion Pressure

*In situ* perfusion of the rat mesentery is a simple and useful method to eliminate the influence of a variety of physiologic influences or homeostatic responses [11]. Rats were anaesthetized with sodium pentobarbital (60 mg/kg, i.p.) (Hospira, Holland). Tracheotomy was performed and catheters were placed in the right and left carotid arteries. Left carotid artery was cannulated for blood pressure measurements using a pressure transducer (Spectramed, South Africa) and a physiograph recorder (Grass, USA). Intact vascular beds were perfused using an extracorporeal circuit and a constant flow Gilson peristaltic pump [12]. Heparin (5 mg/kg) (Hospira, Spain) was then administered by a cannula placed in the left jugular vein. The circuit was established with no interruption of blood flow to the mesenteric bed, pumping from the right carotid artery to the superior mesenteric artery (SMA). The distal portion of the external circuit was connected to a pressure transducer and physiograph recorder. At the beginning of each experiment, flow was adjusted to render the perfusion pressure (PP) equal to the systemic pressure. Flow was kept constant throughout the experiment, changes in the perfusion pressure reflecting the changes in the vascular resistances. The flow rate through the mesenteric vascular bed ranged from 1.5 to 2 ml min^−1^, depending on the animal systemic pressure [12]–[13]. After a 15 min period of stabilization, experiments were performed using six animals to evaluate zolmitriptan (10 mg/kg), propranolol (4.5 mg/kg) or the combination of both. In other set of experiments (N = 5) atenolol (4.5 mg/kg), ICI-118,551 (4.5 mg/kg) or the combination of each one with zolmitriptan were tested. Drugs were dissolved in DMSO and administered intra-arterially via the distal cannula by bolus injection of a maximum of 10 µl using a microsyringe (Exmire, Japan).

### Splanchnic Sympathetic Denervation

Selective chemical sympathectomy (SS) in the splanchnic and peritoneal area was carried out as previously described [14]. A saporin-coupled anti-dopamine-β-hydroxylase antibody (Advance Targeting Systems, USA) was intraperitoneally injected (5 µg/ml in 3 ml of saline) to CCl4-treated rats (N = 4). This is a highly specific noradrenergic damaging agent as it specifically targets cells that express dopamine beta-hydroxylase. Once the immunotoxin has reached the nucleus of the axon, saporin is released and it irreversibly blocks protein synthesis, inducing cell death. Saline alone was given as a control. The efficacy of SS was assessed by measurement of norepinephrine levels in mesenteric organs (spleen, small intestine) with an immunoassay (LDN, Germany).

### *In vitro* Assessment of Mesenteric Artery Contraction

Endothelium-intact mesenteric arterial rings (internal diameter 0.3–0.6 mm) were mounted in a wire myograph in Krebs solution and stretched to an equivalent transmural pressure of 100 mmHg [15]. After equilibration, arteries were mildly depolarized by a threshold concentration of KCl (25 mM) which enhances the contractile responses to zolmitriptan as previously described for other 5HT_1B_ agonists in human mesenteric arteries [16], resembling the *in vivo* responses. In some experiments, arteries were exposed to the β-adrenoceptor agonist isoproterenol (0.1 nM) to mimic the *in vivo* endogenous β-adrenergic sympathetic tone. Then, arteries were exposed to zolmitriptan (10 and 100 µM) followed by propranolol (1 µM) or to propranolol followed by zolmitriptan (N≥5).

### Determination of Cyclic AMP (cAMP) in the Wall of Mesenteric Artery

Under general inhalation anesthesia (isoflurane), healthy rats (N≥4) were injected via a femoral vein with isoproterenol (1 mg/kg) or the same volume of saline. The induction of cAMP with this β-adrenergic agonist allows us to quantify the inhibition of the tested drugs. The other femoral vein was cannulated to inject propranolol (5 mg/kg) or zolmitriptan (10 mg/kg) preceded or not by propranolol injection. Propranolol was infused during 60 seconds and zolmitriptan infusion was stopped after 5 min, when its maximal effect has been described. Rats were sacrificed 6 minutes after the injection and SMA was removed and snapped frozen in liquid nitrogen. Tissues were grounded in a mortar and pestle under liquid nitrogen and tested in accordance with the manufacturer’s instructions (Detect X® Direct Cyclic AMP, Arbor Assays, USA). Protein concentration was determined using the BCA method (Sigma, USA).

### Drug Administration

#### a) Acute experiments

In CCl4-treated or CBDL rats hemodynamic data (PVP, ARF, and PBF) were recorded at baseline and then continuously during and after intravenous infusions of zolmitriptan, propranolol, nadolol and terlipressin. Propranolol and nadolol (Sigma, Spain) were diluted in saline to the final use concentration (5 and 10 mg/kg, respectively). Zolmitriptan (Astra Zeneca, Spain) was diluted in saline and administered at the indicated dose. Terlipressin (Ferring, Spain) was prepared as indicated by the manufacturer and administered at the indicated dose. In the experiments to determine CO and MAP, zolmitriptan (10 mg/kg) and terlipressin (50 µg/kg) were administered as a bolus injection. MAP was continuously recorded while CO determinations were obtained before and after each drug administration, at the point of maximum drug effect (10 and 20 min respectively).

#### b) Chronic experiments

CBDL rats were randomly allocated to two groups that received zolmitriptan (10 mg/kg) or vehicle. Zolmitriptan (Astra Zeneca) was dissolved in citric acid and administered orally by gavage every 12 hours for 7 consecutive days. One hour after the morning dose in day 8, rats were anesthetized, hemodynamic data were obtained and blood samples were collected from the ileocolic vein for biochemical testing. After sacrifice, liver samples were harvested for histochemical analysis in 3.7–4% formaldehyde (Panreac, Spain).

### Laboratory Tests

Blood samples for biochemistry were collected in Vacutainer tubes (BD, UK). After one hour incubation at 37°C, tubes were centrifuged at 2500 RPM for 15 min at RT. Serum was collected and kept at −20°C until analysis. Biochemical parameters were measured using a Cobas Integra Analytical system C311 (Roche, UK).

### Statistics

Data are expressed as mean ± SEM. Statistical significance was determined by Student’s t test or Mann-Whitney test (N<6) for comparisons between two groups. ANOVA test with Bonferroni’s post-test or Kruskal-Wallis test with Dunn’s post’-test (N<6) were used for multiple comparisons. Groups were checked for normality using Shapiro-Wilk test (p≥0.01). Statistical analysis was done using GraphPad Prism 5.0 (GraphPad Software Inc., USA).

## Results

### Administration of Zolmitriptan to CBDL Rats: Effects on PVP, ARF and Systemic Hemodynamics

**Figure 1:**
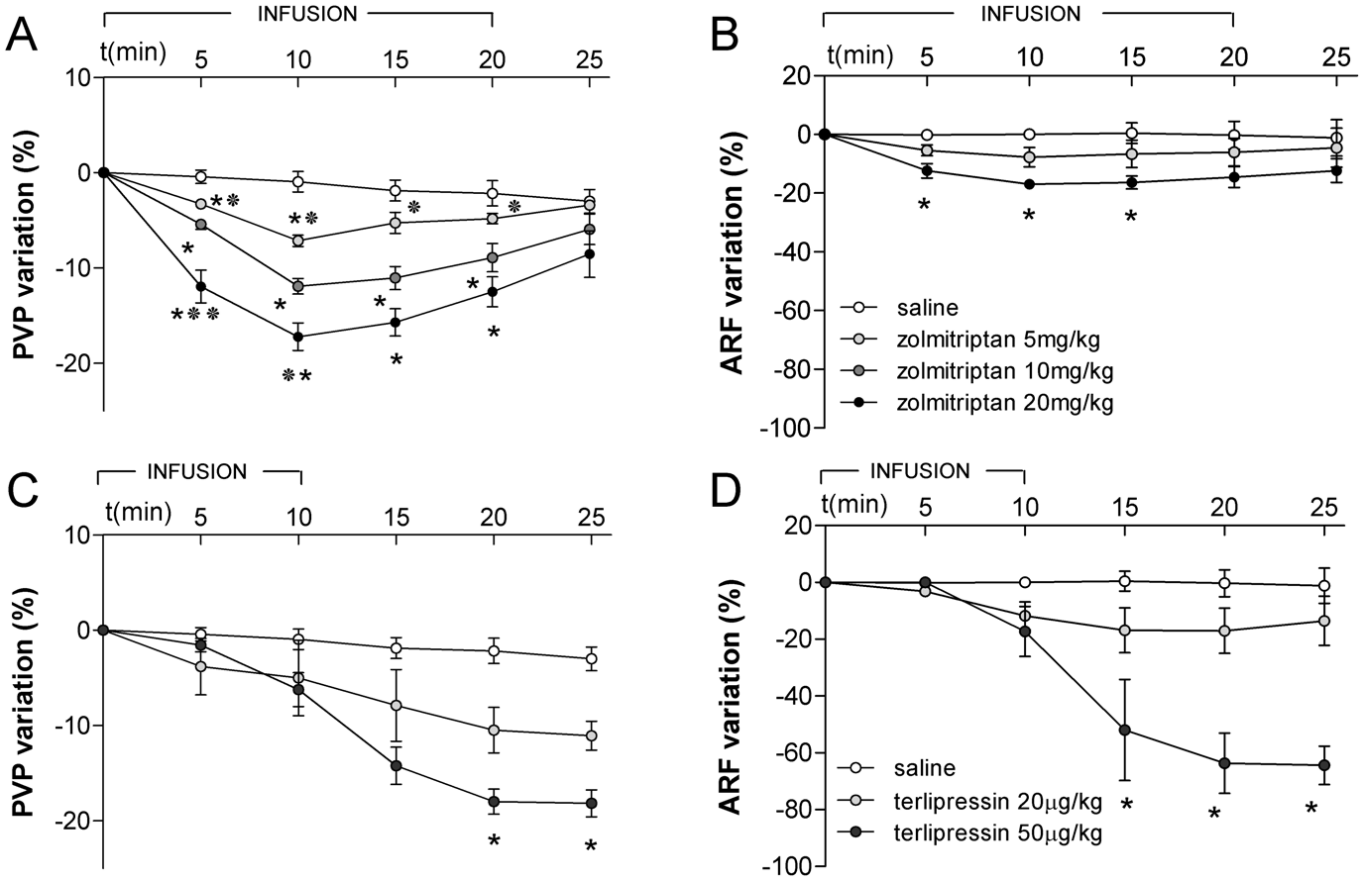
Effect of intravenous infusion of zolmitriptan and terlipressin in CBDL-rats. (A,C) Portal pressure (PVP). (B,D) Renal flow (ARF). *p<0.05 vs saline. */**p<0.05/0.01 vs zolmitriptan dose 10 mg/kg.

Zolmitriptan (at doses of 5, 10 and 20 mg/kg) or saline were infused during 20 min to two weeks-CBDL rats. General characteristics and hemodynamic data of the animals are listed in Table 1. As illustrated in Figure 1, we observed a significant and dose-dependent decrease of PVP in all rats receiving zolmitriptan. With all doses, PVP returned to baseline 5 min after the end of the infusion (Figure 1A). Zolmitriptan caused similar effects on PVP in four weeks-CBDL rats (Figure S1).

**Table 1:** Body weight, biochemical data and basal hemodynamic parameters of rats with portal hypertension which received i.v. infusion of zolmitriptan, terlipressin or saline.

	CCl4 model		CBDL model		
	Saline (N = 4)	Zolmitriptan (N = 6)	Saline (N = 4)	Zolmitriptan (N = 14)	Terlipressin (N = 7)
Body weight (g)	402 (360–489)	443 (393–483)	383 (331–416)	381 (275–408)	407 (307–438)
ALP (U/L)	115 (80–244)	163 (115–289)	653 (561–677)	476 (318–753)	433 (82–1399)
ALT (U/L)	44 (21–52)	56 (44–108)	255 (200–356)	209 (104–460)	145 (8–329)
bilirubin (mg/dL)	0.2 (0.1–0.6)	0.2 (0.1–0.4)	15.4 (12.3–18.4)	11.5 (9–17)	10.1 (0.2–15)
albumin (g/dL)	3.2 (3–3.4)	3 (2.6–3.2)	4 (3.1–4.1)	2.9 (2.5–4.1)	3 (0.6–4)
Hemodynamic data					
PVP (mm Hg)	11.6±0.9	12.7±0.5	13.1±0.6	14.1±0.5	13.8±0.7
Renal flow (ml/min)	6.4±1.3	7.0±0.6	5.4±0.6	4.2±0.6	4.2±0.4
heart rate (beats/min)	325±12.3	332±17.4	330±10.5	335±10.81	332±20

Values of body weight and biochemical variables are expressed as median and ranges and hemodynamic data are expressed as mean±SEM. ALP: alkaline phosphatase, ALT: alanine transaminase, AST: aspartate aminotransferase, PVP: portal vein pressure.

**Figure 2:**
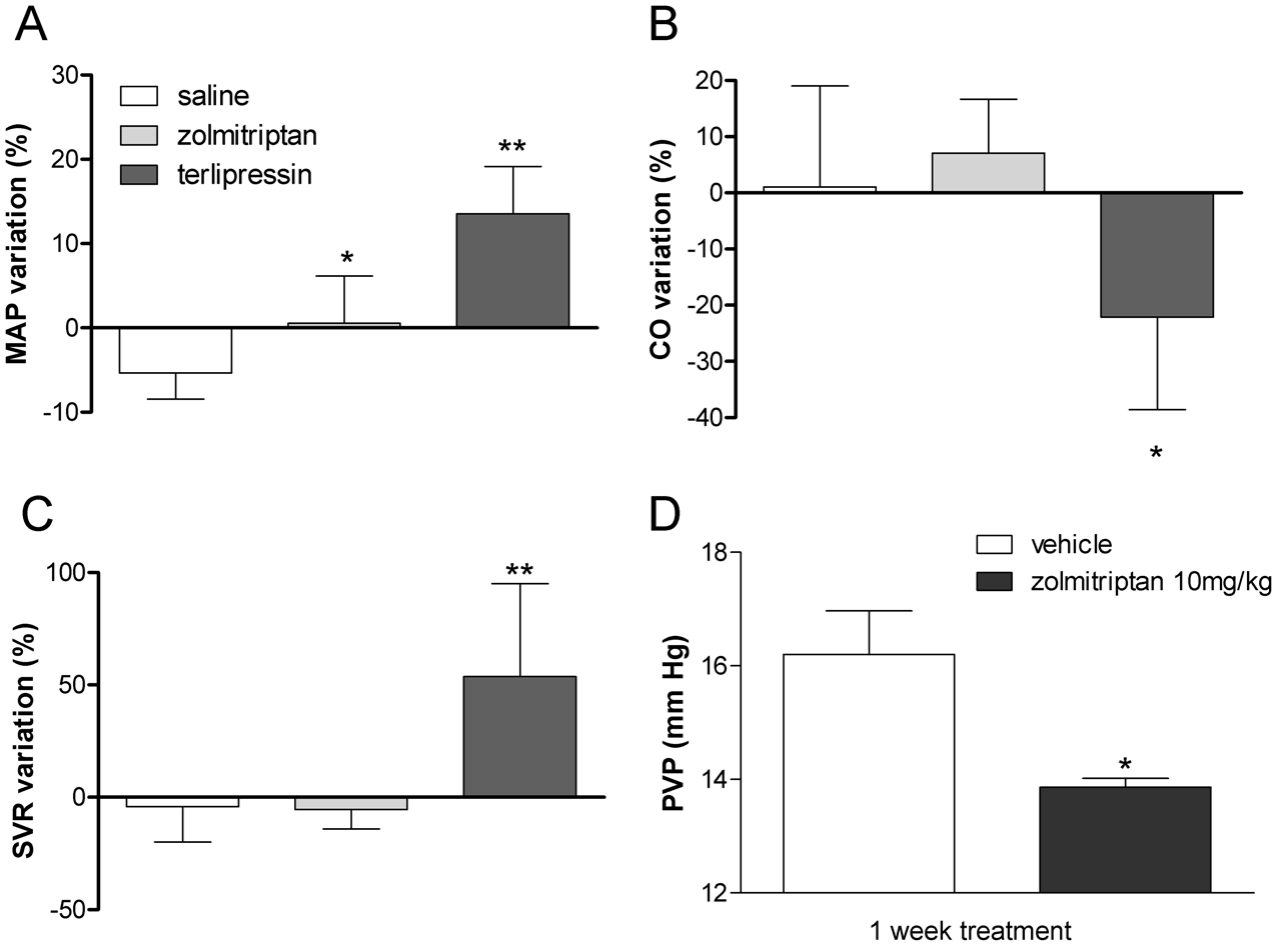
Systemic and chronic effects of zolmitriptan and terlipressin in CBDL-rats. Measurement of arterial pressure (MAP) (A), cardiac output (CO) (B) and systemic vascular resistance (SVR) (C) after zolmitriptan, terlipressin or saline administration. (D) Effect of chronic administration of zolmitriptan in portal pressure (PVP). */**p<0.05/0.01 vs saline.

We also found that zolmitriptan caused a slight and transient decrease in ARF (Figure 1B) which was significantly less intense than that produced by doses of terlipressin that induced similar effects on PVP (maximal reduction of ARF 17% vs 64%, p<0.05) (Figure 1C and D). Moreover, in rats subjected to four week-CBDL, we found that terlipressin (50 µg/kg) provoked a substantial increase of MAP (14% with respect baseline) together with a significant decrease of CO and a marked elevation of SVR while zolmitriptan caused no significant changes in CO and SVR and only a mild elevation of MAP (1% with respect baseline) (Figure 2A, B and C).

To see whether maintenance therapy with zolmitriptan could reduce PVP in rats with two weeks-CBDL, animals were randomly allocated (N = 7 per group) to receive zolmitriptan (10 mg/kg) or vehicle for 7 days. Two rats from each group died before sacrifice and hemodynamic measurement failed for technical reasons in one rat. We found that animals treated with zolmitriptan experienced a decrease in PVP of 14% compared to those receiving vehicle (p<0.05) (Figure 2D). No relevant changes in biochemical parameters were found in association with this therapy (Table 2).

**Table 2:** Biochemical data and hemodynamic parameters in CBDL rats after chronic treatment with zolmitriptan or vehicle.

	CBDL model		
	vehicle (N = 5)	zolmitriptan (N = 5)	
alkaline phosphatase (U/L)	415.4±12.0	481.8±133	
alanine transaminase (U/L)	92.6±3.2	91.6±18.9	
aspartate aminotransferase (U/L)	398.4±11.8	410±118.1	
bilirubin (mg/dL)	12.1±0.3	10.3±0.6	
albumin (g/dL)	3.1±0.1	2.6±0.1 a	
Hemodynamic data			
portal pressure (mm Hg)	16.2±0.8	13.9±0.2 a	

Data are expressed as mean±SEM. _a_ compared to the vehicle group, p<0.05.

### Portal Hypotensive Effects of Zolmitriptan in Rats with CCl_4_-induced Liver Cirrhosis: Effect of Splanchnic Sympathectomy (SS)

**Figure 3:**
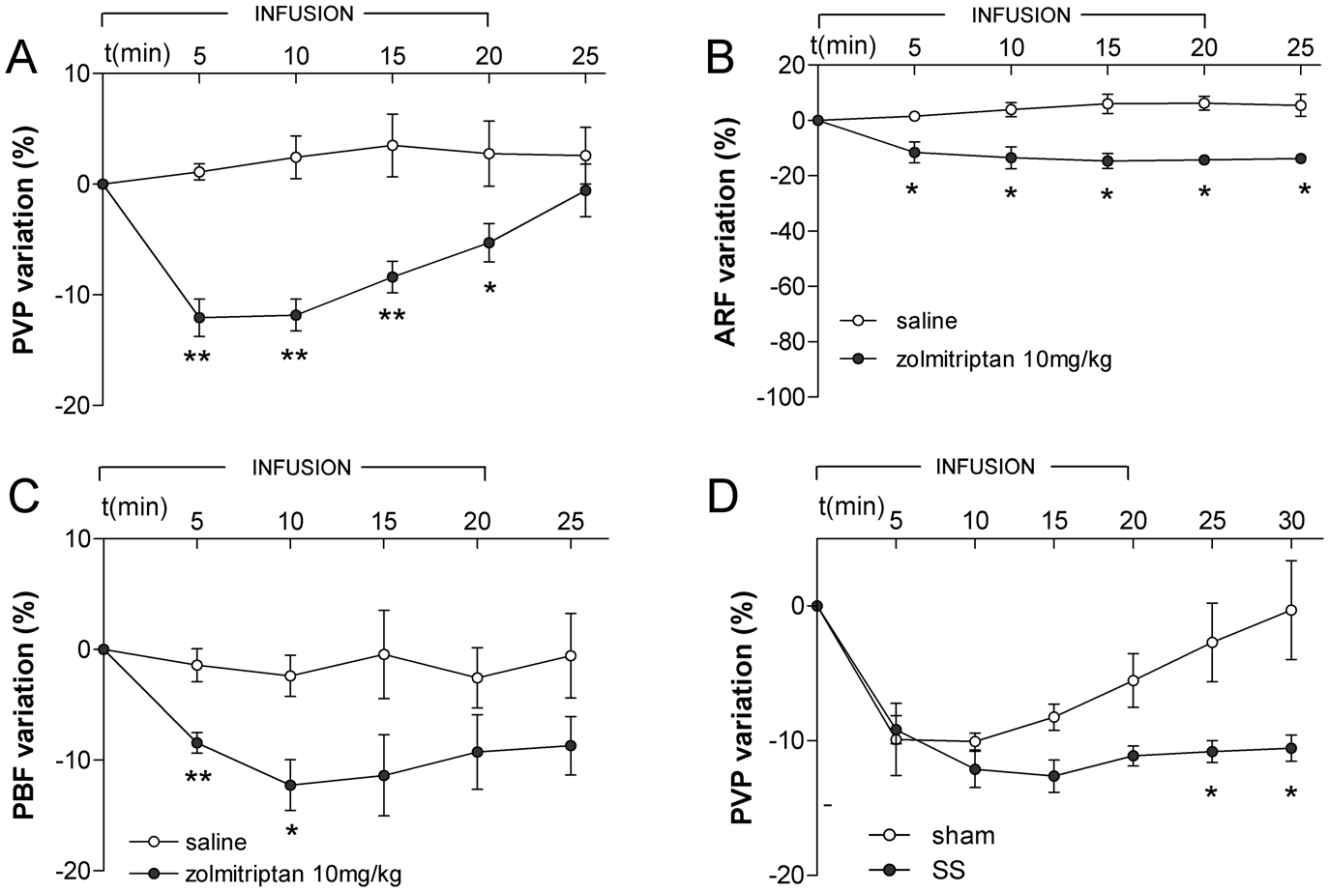
Effect of intravenous infusion of zolmitriptan in CCl4-treated rats. (A) Portal pressure (PVP). (B) Renal flow (ARF). (C) Portal flow (PBF). (D) Portal pressure after SS. */** significance vs saline or sham p<0.05/0.01.

To analyze if the effect of zolmitriptan on portal hypertension was model-dependent we also evaluated the effect of this drug in rats with CCl4-induced liver cirrhosis. Animals were divided into two groups which received an intravenous infusion (20 min) of saline or zolmitriptan (10 mg/kg). General characteristics and hemodynamic data of the experimental groups are listed in Table 1. As shown in Figure 3A, zolmitriptan caused a significant reduction in PVP as compared to rats receiving saline. As in the CBDL model, the decrease of PVP was rapid (maximum descent at 5 min) but reverted to basal values when the infusion was discontinued. The reduction of PVP was accompanied by a slight but statistically significant decrease in ARF (Figure 3B) from 7±1.4 ml/min at baseline to 5.9±1.2 ml/min at the end of the infusion (p<0.05). HR did not differ significantly between the two groups (319±20 bpm vs 322±20 bpm). MAP remains quite stable in this model of portal hypertension, even at high doses (20 mg/kg), with only a slight transient decrease of its values at the initiation of the infusion (Figure S2). Also, we observed that zolmitriptan caused a diminution of PBF (Figure 3C) which paralleled the descent in PVP suggesting that this drug reduces portal blood inflow by promoting mesenteric artery vasoconstriction.

As noted above, the fall in PVP that occurs in cirrhotic rats treated with zolmitriptan reverts soon after discontinuing drug infusion. To determine whether sympathetic innervation might counteract the splanchnic vasoconstrictive activity of zolmitriptan we infused this drug in two groups of rats with CCl4-induced liver cirrhosis, of which one was subjected to SS, while the other group received saline instead (Figure S3). We found that in the control group PVP was reduced during zolmitriptan infusion but reverted to baseline upon cessation of drug infusion while in the group subjected to SS the descent of PVP persisted during all the study period (Figure 3D).

### NSBBs Prolong and Enhance the Portal Hypotensive Effect of Zolmitriptan in Rats with CCl4-induced Liver Cirrhosis

**Figure 4:**
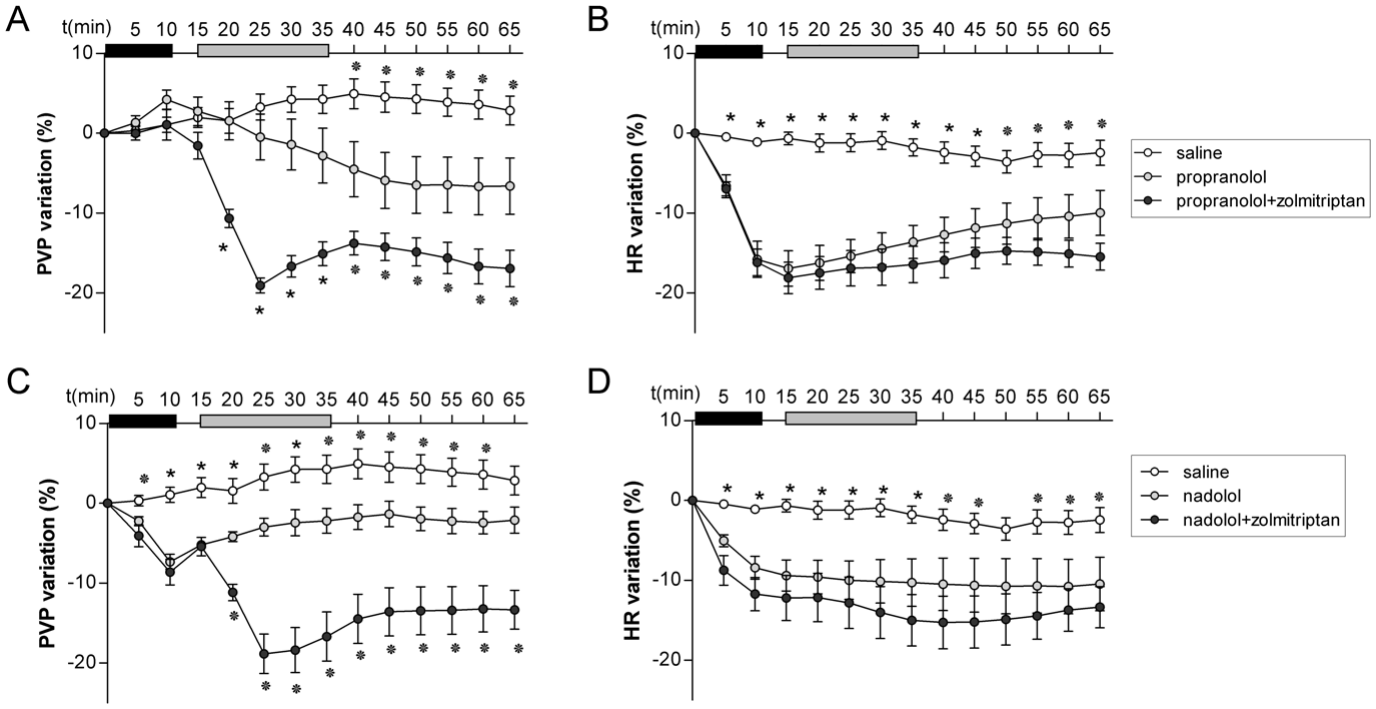
Effect of zolmitriptan in combination NSBB in CCl4-treated rats. Propranolol or nadolol were administered as an intravenous infusion and 5 minutes later, zolmitriptan or saline infusion were started and portal pressure (PVP) (A,C) and heart rate (HR) (B,D) recorded. Infusion time is indicated with black (beta-blockers) and grey bars (zolmitriptan). */* indicates significance vs rats receiving only NSBB (p<0.05/0.01).

Propranolol, a NSBB which is the cornerstone therapy for the prevention of variceal bleeding, lowers PVP by blocking β1- and β2-receptors. The effect of β1-blockade, which reduces CO, appears to be less relevant than the effect of β2-blockade which promotes splanchnic vasoconstriction by preventing vasodilation [17]. Since zolmitriptan induces splanchnic vasoconstriction, it is reasonable to hypothesize that this drug may cooperate with propranolol in lowering PVP. To analyze the interaction of zolmitriptan and NSBB, we infused zolmitriptan (10 mg/kg during 20 min) after a 10-min infusion of propranolol or nadolol to rats with CCl4-induced liver cirrhosis (general features and hemodynamic data of the animals are presented in Table S1). We used the model of CCl4-induced liver cirrhosis since it was reported that propranolol has no hemodynamic effects in CBLD rats [18]. As expected, propranolol and nadolol produced a significant reduction in HR (−15% and −8% respectively, p<0.01) and PVP (−6%, p<0.05 and −7%, p<0.01, respectively) when compared to saline (Figure 4 A and C). The combination of propranolol or nadolol with zolmitriptan resulted in a significant decrease in PVP (−16%, p<0.01) compared to rats receiving NSBB alone. Interestingly, the reduction of PVP achieved by combining NSBB and zolmitriptan was more durable and persisted beyond drug infusion. This is in contrast with the transient fall in PVP observed when zolmitriptan was administered alone (see Figure 3). Of note, the drop in HR did not differ significantly between groups receiving NSBB or the combination therapy (Figure 4B and D).

### Effect of Zolmitriptan Alone or in Combination with NSBB on Superior Mesenteric Artery Perfusion Pressure (SMA-PP)

**Figure 5:**
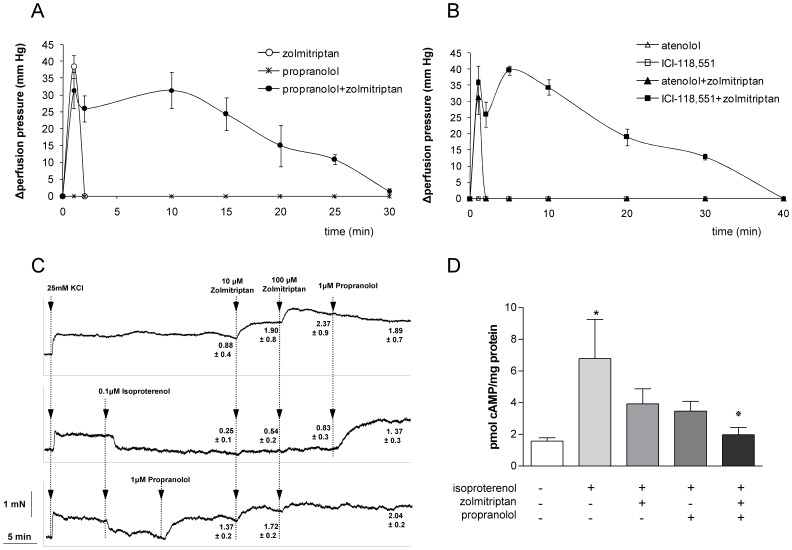
Effects of zolmitriptan in combination with beta-blockers on splanchnic vasculature of healthy rats. (A,B) Zolmitriptan alone or in combination with propranolol, atenolol and ICI-118,551 were administered intra-arterially and perfusion pressure recorded. (C) *In vitro* mesenteric artery contraction studies after zolmitriptan and propranolol administration in the presence or absence of isoproterenol. (D) cAMP measurement in mesenteric artery wall after administration of zolmitriptan, propranolol or the combination or both in the presence of isoproterenol. */*p<0.05 vs control group/group receiving only isoproterenol.

To further analyze the interaction between propranolol and zolmitriptan on mesenteric artery vasoconstriction, we evaluated the SMA-PP in healthy rats treated with the drugs. In previous experiments we observed that the decrease in SMA blood flow was similar in cirrhotic and healthy rats indicating that liver cirrhosis does not decrease the sensitivity of the splanchnic vasculature to the vasoconstrictive effects of zolmitriptan. After intra-arterial administration of zolmitriptan (10 mg/kg), propranolol or the combination of both, we observed that unlike propranolol or vehicle, zolmitriptan induced a marked but short lasting (about 1 minute) increase in SMA-PP. Interestingly, when zolmitriptan was given in combination with propranolol, the increase in PP was similar but lasted longer (nearly 30 min) (Figure 5A). In order to discern the role of each beta-adrenergic receptor in this vasoconstrictor effect, we measured SMA-PP after injection of specific β1- (atenolol) and β2-(ICI-118,551) blockers and after the combined administration of each one of them with zolmitriptan (Figure 5B). None of the beta-blockers modified PP when injected alone. Interestingly, while atenolol did not change the contractile response to the triptan, the joint administration of zolmitriptan with the β2-blocker resulted in a prolonged increase in PP comparable to that seen after administration of zolmitriptan plus propranolol.

### Effect of Zolmitriptan in Combination with NSBB on Catecholamine-induced Relaxation of Mesenteric Artery *in vitro*

In preliminary experiments, we observed that zolmitriptan produced a weak and variable contractile response in isolated mesenteric arteries but when arteries were exposed to 25 mM KCl, the triptan induced a dose-dependent contractile response (Figure 5C, upper graph). Propranolol had no apparent effect on vascular tone, due to the absence of sympathetic tone in these *in vitro* experiments. The β-adrenoceptor agonist isoproterenol strongly relaxed KCl-induced contraction and inhibited the contraction promoted by zolmitriptan, a response which was restored by subsequent addition of propanolol to the incubation medium (Figure 5C, middle graph). In another set of experiments, pre-incubation of the SMA with propranolol reversed the relaxation induced by isoproterenol and facilitated the contractile response to zolmitriptan (Figure 5C, lower graph). Altogether, these experiments indicate that β2-receptor activation attenuates zolmitriptan-induced SMA vasoconstriction and that this inhibitory effect is reversed by propranolol.

### Effect of Zolmitriptan in Combination with NSBB on cAMP Levels in the Wall of Mesenteric Artery

β2-receptor activation increases intracellular cAMP levels which mediate relaxation of smooth muscle cells of the arterial wall [19]. Thus, we decided to analyze the effect of zolmitriptan, propranolol and the combination of the two drugs on cAMP levels in the mesenteric artery wall. To this aim rats received a bolus of isoproterenol, followed by an intravenous injection of propranolol or an infusion of zolmitriptan preceded or not by propranolol injection. A group receiving saline served as control. Animals were sacrificed 6 minutes after treatment and mesenteric artery was sampled for cAMP determinations. Isoproterenol promoted a significant increase (p<0.05) of cAMP levels in the arterial wall and both zolmitriptan and propranolol tended to attenuate this effect but the differences with isoproterenol alone were not statistically significant. However the combination of the two drugs showed an additive effect that caused a more intense and statistically significant (p<0.05) reduction of cAMP in the SMA wall compared to isoproterenol alone (Figure 5D).

## Discussion

Zolmitriptan is a selective serotonin 5-HT_1B/1D_ receptor agonist with proven benefit in the treatment of migraine as result of its cranial vasoconstrictive effect. [20]. Several clinical trials have confirmed the efficacy and favorable safety profile of this drug [21]. In addition to acting on cranial vessels, zolmitriptan also causes peripheral vasoconstriction in specific territories. Of particular interest when considering possible therapies for portal hypertension is the fact that 5HT_1B/D_ receptors abound in human SMA where they mediate robust vasoconstrictive effects [6] [22]. Therefore we have tested zolmitriptan as a therapeutic option for subjects with portal hypertension, since drugs able to reduce blood inflow into the splanchnic area could be potentially useful to reduce the risk of bleeding from esophageal varices in these patients and to treat variceal hemorrhage.

In the present study we show that zolmitriptan causes a dose-dependent reduction of PVP in rats with portal hypertension. This effect is associated with a parallel reduction of PBF accompanied by increased mesenteric vascular resistance, indicating that vasoconstriction of splanchnic arteries is a key mechanism implicated in the reduction of PVP. Zolmitriptan has several advantages in the treatment of patients with portal hypertension including its modest influence on MAP and ARF (even at high doses) which is not comparable to the effects of terlipressin. Our results with zolmitriptan in experimental animals are in accordance with pharmacological studies in humans where no significant changes in MAP have been described [23]. Also, previous studies indicate that sustained zolmitriptan administration does not cause tachyphylaxis [24]–[25]. In agreement with these data we observed 14% reduction of PVP in CBDL rats treated with zolmitriptan for one week. Moreover there are reports of its good tolerability after repeated administration in patients [26] [23].

The portal hypotensive effect of zolmitriptan is transient as PVP returns to basal values rapidly upon interruption of drug infusion. Our study indicates that adrenergic drive opposes the splanchnic vasoconstrictive effect of zolmitriptan. By performing SS we showed that elimination of sympathetic impulse augmented and prolonged the reduction in PVP induced by zolmitriptan. Moreover our *in vitro* experiments demonstrated that SMA-contraction induced by zolmitriptan was abrogated by isoproterenol and restored by addition of propranolol. Accordingly, we found that combining zolmitriptan and propranolol reduced PVP to a greater extent and also for a longer period of time. Prolongation of the vasoconstrictive effect of zolmitriptan by concurrent administration of NSBB was also observed by measuring PP following injection of zolmitriptan, propranolol or both into the SMA. In these studies propranolol had no effect on the PP while zolmitriptan caused an abrupt but brief elevation of this parameter. Importantly, the injection of both drugs resulted in a marked prolongation of zolmitriptan vasoconstrictive effect. Interestingly, this additive effect was achieved even at a low dose of propranolol. Low dose of NSBB causes unopposed splanchnic vasoconstriction, leading to reduced PBF and, consequently, decreased portal hypertension. As the dose is increased, PBF is further reduced by the negative effect of NSBB on CO [27].

It has been reported that propranolol inhibits the metabolism of zolmitriptan [28]. Thus, we tested if other beta-blocker devoid of metabolic interference, such as nadolol [29], used in combination with zolmitriptan acted similarly on PVP. Our data showed that the decrease in PVP was similar when zolmitriptan was combined with propranolol or with nadolol. These results suggest that the enhancement of the portal hypotensive effects observed when triptans and NSBB are given in combination are due to pharmacological interaction of the two drugs rather than changes in drug metabolism. To further explore the mechanism of this interaction we used selective β1- and β2-blockers in combination with zolmitriptan. We found that β2-adrenoceptor blocking activity was responsible for propranolol synergy with zolmitriptan. Unlike β1-receptors almost exclusively present in the heart, β2-receptors are located in blood vessels. These receptors are abundant in the splanchnic vasculature where they cause vasodilatation mediated by cAMP [19]. It is well documented that cirrhosis is associated with high levels of circulating catecholamines and elevated cAMP production in the splanchnic circulation [30]. These changes contribute to increase portal vein inflow and PVP. In the present study we found that activation of 5HT1B/D receptors with zolmitriptan and inhibition of beta-adrenergic receptors with propranolol cooperate in reducing cAMP in the wall of SMA and therefore in attenuating the decrease in splanchnic vascular resistances present in liver cirrhosis. This is consistent with the canonical signaling pathways of these two G-coupled receptors which involve the inhibition and activation, respectively, of cAMP synthesis by adenylyl cyclase [19] [31].

Propranolol is the cornerstone of pharmacological treatment of portal hypertension but only 37% of patients are hemodynamic responders [4]. Our findings suggest a potential use of zolmitriptan in combination with β-blockers in the pharmacological treatment of portal hypertension particularly for patients who are refractory to beta-blockers. Interestingly, patients with frequent migraine attacks receiving triptans have been treated with beta-blockers in order to obtain better control of migraine [29]. A similar paradigm could be proposed for portal hypertension.

In conclusion, our data give support for future clinical trials directed to test the effect of combination therapy of NSBB and triptans in the prevention of variceal bleeding in cirrhotic patients with portal hypertension.

## Supporting Information

Figure S1 **Effect of zolmitriptan in PVP (A) and ARF (B) in 4 week-CBDL rats (N = 4 per group).** Zolmitriptan (10 mg/kg) was administered through the femoral vein catheter as an infusion (20 min). Values are presented as mean±SEM. *p<0.05 vs saline. PVP: portal venous pressure, ARF: arterial renal flow.
(PDF)

Figure S2 **Effect of zolmitriptan in PVP (A), ARF (B), HF (C) and MAP (D) in CCl4-treated rats (N = 5 per group).** Zolmitriptan (20 mg/kg) was administered through the femoral vein catheter as an infusion (20 min). Values are presented as mean±SEM. */**p<0.05/0.01 vs saline. PVP: portal venous pressure, ARF: arterial renal flow, HF: heart frequency, MAP: mean arterial pressure.
(PDF)

Figure S3 **Levels of norepinephrine in the spleen (A) and duodenum (B) of CCl4-treated rats subjected to either sham or splanchnic sympathectomy (SS).** *p<0.05 vs sham.
(PDF)

Table S1 **Body weight, biochemical data and basal hemodynamic parameters of rats with portal hypertension which received i.v. infusion of beta-blockers or saline alone or beta-blockers followed by zolmitriptan.** Body weight and biochemical parameters are expressed as median and ranges. Hemodynamic data are expressed as mean±SD. prop: propranolol, zolm: zolmitriptan, ALP: alkaline phosphatase, ALT: alanine transaminase, AST: aspartate aminotransferase, PVP: portal vein pressure, ARF: arterial renal flow.
(PDF)
