# Supplementary figures and images for: Zolmitriptan: A Novel Portal Hypotensive Agent Which Synergizes with Propranolol in Lowering Portal Pressure
Source: PLoS One. 2013 Jan 16;8(1):e52683. doi: 10.1371/journal.pone.0052683 (PMC3547109; doi:10.1371/journal.pone.0052683)

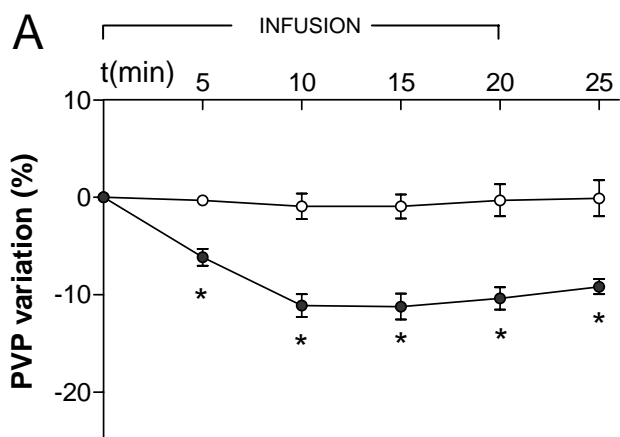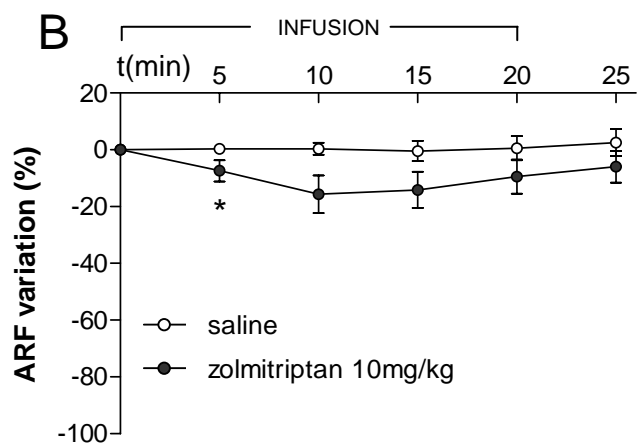

Supplement: Figure S1 — Effect of zolmitriptan in PVP (A) and ARF (B) in 4 week-CBDL rats (N = 4 per group). Zolmitriptan (10 mg/kg) was administered through the femoral vein catheter as an infusion (20 min). Values are presented as mean±SEM. *p<0.05 vs saline. PVP: portal venous pressure, ARF: arterial renal flow. (PDF) [file pone.0052683.s001.pdf]

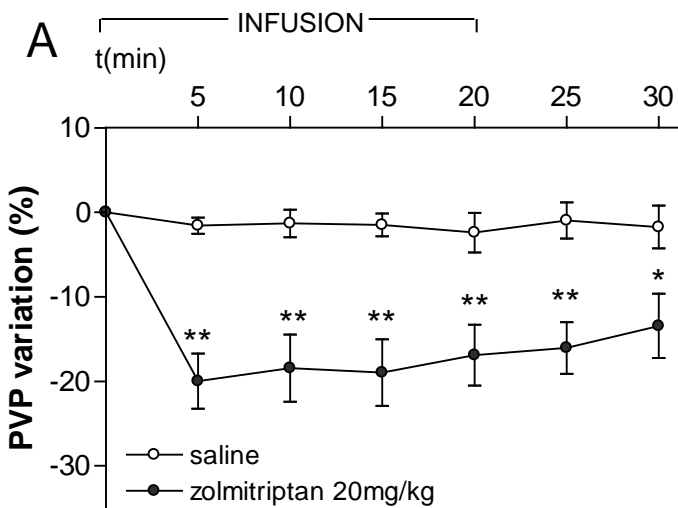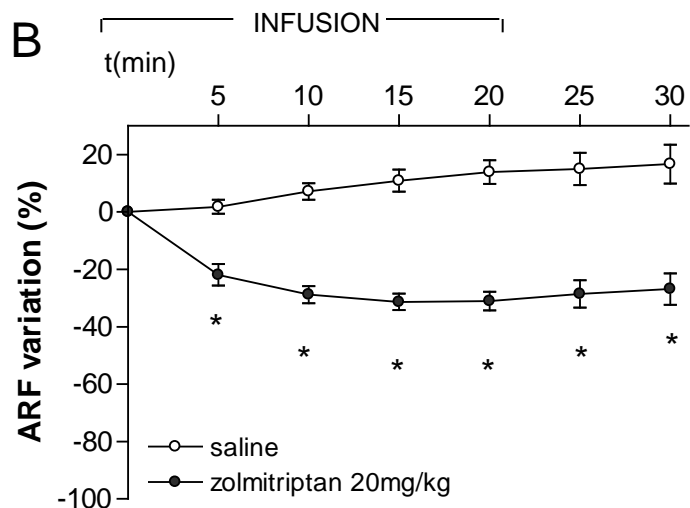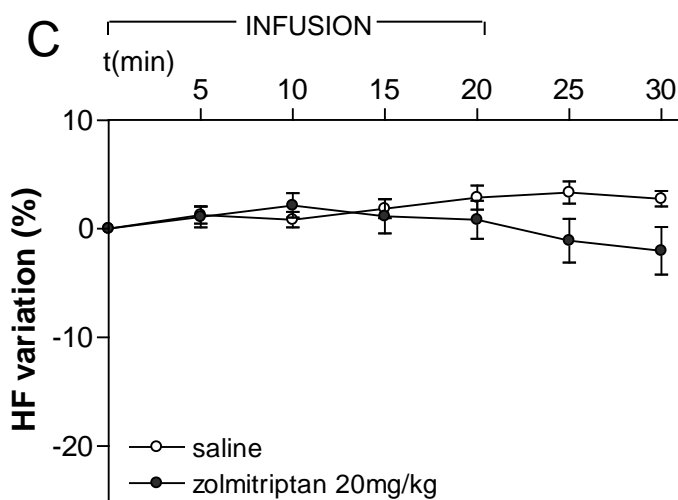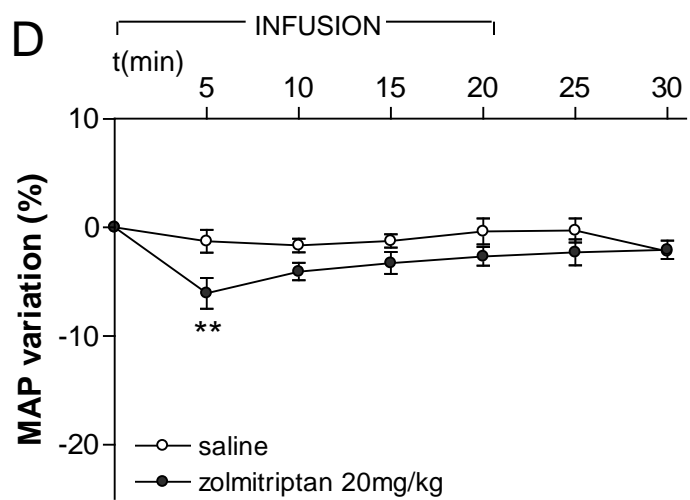

Supplement: Figure S2 — Effect of zolmitriptan in PVP (A), ARF (B), HF (C) and MAP (D) in CCl4-treated rats (N = 5 per group). Zolmitriptan (20 mg/kg) was administered through the femoral vein catheter as an infusion (20 min). Values are presented as mean±SEM. */**p<0.05/0.01 vs saline. PVP: portal venous pressure, ARF: arterial renal flow, HF: heart frequency, MAP: mean arterial pressure. (PDF) [file pone.0052683.s002.pdf]

A

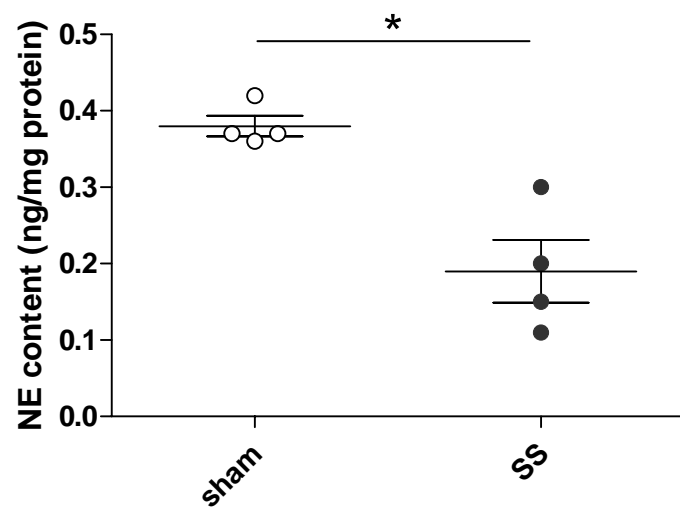

B

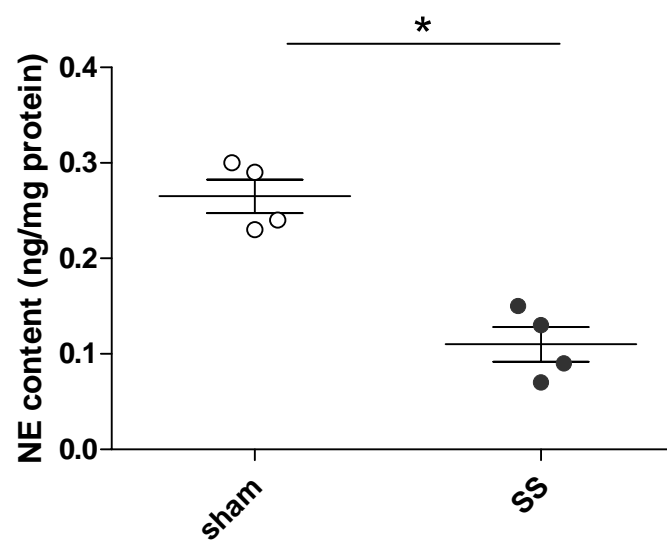

Supplement: Figure S3 — Levels of norepinephrine in the spleen (A) and duodenum (B) of CCl4-treated rats subjected to either sham or splanchnic sympathectomy (SS). *p<0.05 vs sham. (PDF) [file pone.0052683.s003.pdf]
