# Supplementary material for: Zolmitriptan: A Novel Portal Hypotensive Agent Which Synergizes with Propranolol in Lowering Portal Pressure
Source: PLoS One. 2013 Jan 16;8(1):e52683. doi: 10.1371/journal.pone.0052683 (PMC3547109; doi:10.1371/journal.pone.0052683)
Supplement: Table S1 — Body weight, biochemical data and basal hemodynamic parameters of rats with portal hypertension which received i.v. infusion of beta-blockers or saline alone or beta-blockers followed by zolmitriptan. Body weight and biochemical parameters are expressed as median and ranges. Hemodynamic data are expressed as mean±SD. prop: propranolol, zolm: zolmitriptan, ALP: alkaline phosphatase, ALT: alanine transaminase, AST: aspartate aminotransferase, PVP: portal vein pressure, ARF: arterial renal flow. (PDF) [file pone.0052683.s004.pdf]

Table S1. Body weight, biochemical data and basal hemodynamic parameters of rats with portal hypertension which received i.v. infusion of beta-blockers or saline alone or beta-blockers followed by zolmitriptan.

	Saline (N=9)	Prop (N=8)	Prop+Zolm (N=9)	Nadolol (N=4)	Nadolol+zolm (N=4)
Body weight (g)	382 (308-465)	356 (335-444)	372 (222-413)	379 (336-410)	380 (350-418)
ALP (U/L)	75 (57-303)	153 (104-302)	123 (99-344)	158 (62-288)	111 (88-147)
ALT (U/L)	60 (30-89)	51 (10-270)	39 (17-165)	56.5 (35-82)	19 (6-41)
Bilirubin (mg/dL)	0.1 (0.1-0.8)	0.3 (0.1-0.7)	0.2 (0.1-0.9)	0.3 (0.1-0.6)	0.2 (0.1-0.3)
Albumin (g/dL)	3.1 (1.5-3.6)	2.8 (2.3-3)	2.8 (2.1-3.3)	3.0 (2.5-3.6)	3.1 (2.8-3.5)
Hemodynamic data					
PVP (mm Hg)	12.8±2.3	14.3±1.3	13.5±1.5	14.6±2.1	15.7±3.5
ARF (ml/min)	5.3±1.1	4.7±1.1	5.2±1.3	7.1±1.5	7.2±2.7
Heart rate (beats/min)	329±18	324±20	325±27	308±8	317±22

Body weight and biochemical parameters are expressed as median and ranges. Hemodynamic data are expressed as mean±SD. prop: propranolol, zolm: zolmitriptan, ALP: alkaline phosphatase, ALT: alanine transaminase, AST: aspartate aminotransferase, PVP: portal vein pressure, ARF: arterial renal flow.
